# Supplementary material for: Comparison of endogenously expressed fluorescent protein fusions behaviour for protein quality control and cellular ageing research
Source: Sci Rep. 2021 Jun 17;11:12819. doi: 10.1038/s41598-021-92249-1 (PMC8211707; doi:10.1038/s41598-021-92249-1)
Supplement: Supplementary file 2 — Supplementary Information 1. [file 41598_2021_92249_MOESM2_ESM.pdf]

Comparison of endogenously expressed fluorescent protein fusions behaviour for protein quality control and cellular aging research

Kara L Schneider[1], Adam JM Wollman[2], Thomas Nyström[1], Sviatlana Shashkova[1]

[1]Department of Microbiology and Immunology, Institute of Biomedicine, Sahlgrenska Academy, University of Gothenburg, 405 30 Gothenburg, Sweden

[2] Newcastle University Biosciences Institute, Newcastle NE2 4HH, United Kingdom.

Correspondence to: Sviatlana Shashkova, sviatlana.shashkova@gu.se

Supplementary figure 1.

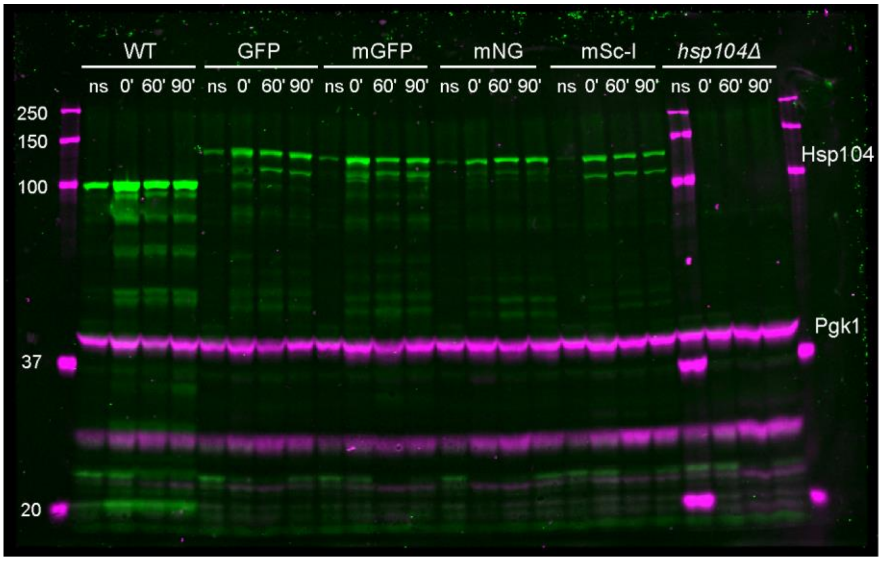

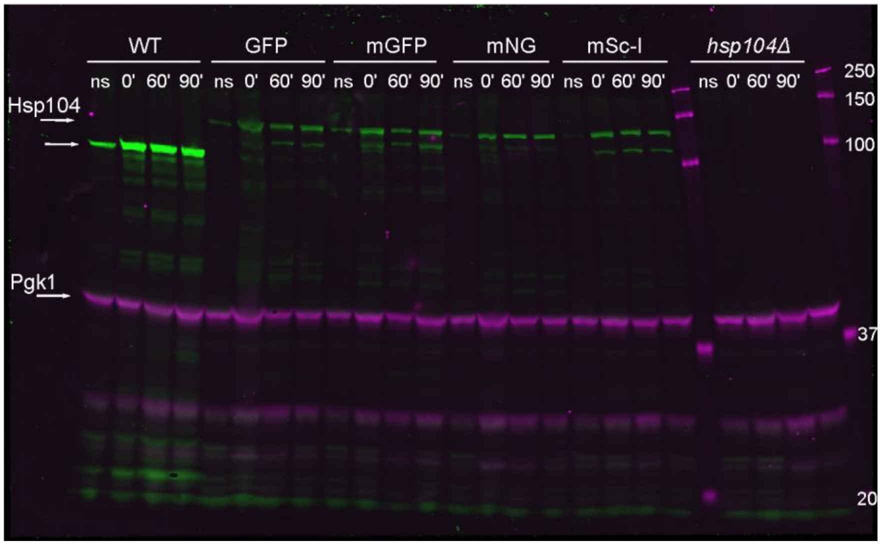

Western blot images. Hsp104 protein expression levels from total protein extracts before (ns) and right after (0’) to the heat stress as well as 60 and 90 min after recovery at 30°C, 180 rpm, in the wild type (WT) strain and cells expressing Hsp104 tagged with GFP, mGFP, mNeonGreen (mNG) and mScarlet-I (mSc-I). The *hsp104Δ* strain is used as a negative control. Bands detected with anti-Hsp104 antibodies, 800 nm channel, (green) and anti-Pgk1 (magenta), 700 nm channel. The molecular weight marker is visible in the 700 nm channel. For the ease of interpretation, we merged both images. Molecular sizes of the proteins correspond to: Hsp104 102 kDa, fluorescent fusions of Hsp104 129 kDa, Pgk1 45 kDa.

Supplementary figure 2.

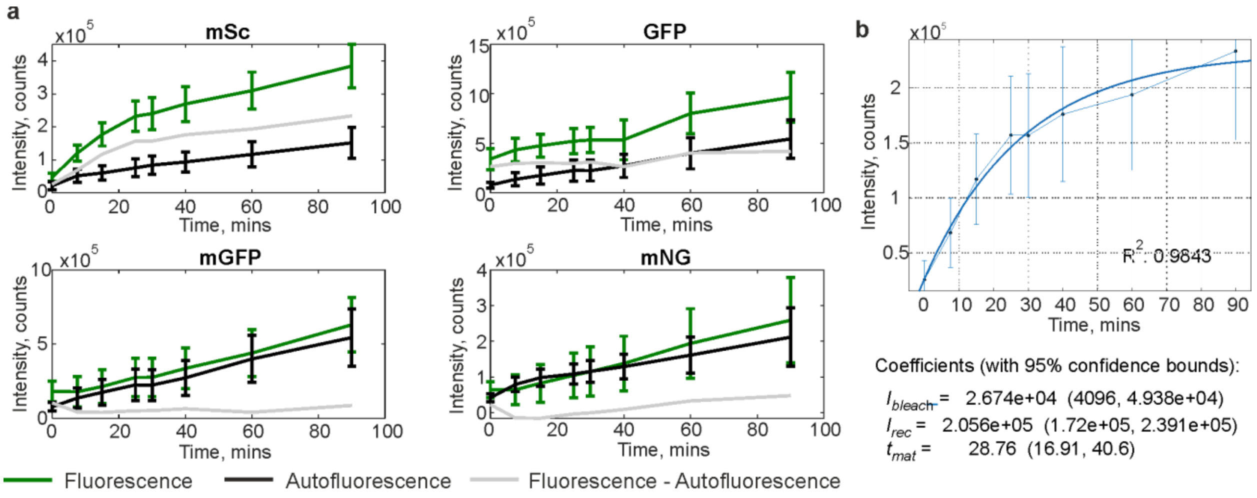

a. Fluorescence recovery after complete bleaching. Fluorescence signal (green) was measured at 0, 7.5, 15, 25, 30, 40, 60 and 90min after cells expressing fluorescent Tom70 fusions were completely bleached. Autofluorescence (black) was measured as any fluorescent signal detected from the unlabelled wild type strain in the appropriate channel. Any maturation of the fluorescent protein is estimated as a fluorescence signal above autofluorescence (grey). **b.** Estimation of the maturation time of the mScarlet-I within the Tom70 fusion.

Supplementary Movie 1.

Representative video of cells expressing Hsp104-mSc-I (red spots) recovering after the heat stress (0 -90 min, 5 min acquisition time intervals). Scale bar 2 µm.
